# How do Brazilian citizens perceive animal welfare conditions in poultry, beef, and dairy supply chains?

**DOI:** 10.1371/journal.pone.0202062

**Published:** 2018-12-19

**Authors:** Ricardo Guimarães de Queiroz, Carla Heloisa de Faria Domingues, Maria Eugênia Andrighetto Canozzi, Rodrigo Garófallo Garcia, Clandio Favarini Ruviaro, Júlio Otávio Jardim Barcellos, João Augusto Rossi Borges

**Affiliations:** 1 Federal University of Grande Dourados, Dourados, Mato Grosso do Sul, Brazil; 2 National Institute of Agricultural Research (INIA), La Estanzuela, Uruguay; 3 Centro de Estudos e Pesquisas em Agronegócios, Federal University of Rio Grande do Sul, Porto Alegre, Rio Grande do Sul, Brazil; Tokat Gaziosmanpasa University, TURKEY

## Abstract

The aim of this study was to understand the perceptions of Brazilian citizens about the general conditions of animal welfare in the poultry, beef, and dairy supply chains. To reach this aim, an online survey was conducted. The analysis was based on descriptive statistics and three logistic regression models. Results of descriptive statistics showed that citizens in Brazil had mostly negative perceptions about the conditions of animal welfare in the poultry, beef, and dairy supply chains. Results of the logistic regression models showed that citizens with a background in agricultural/veterinary sciences, and citizens who reported a higher level of knowledge about poultry and dairy supply chains were more likely to perceive the general conditions of animal welfare in these two supply chains as being bad. Citizens who reported previous contact with poultry farms were also more likely to perceive the general conditions of animal welfare in the poultry supply chain as being bad. In addition, the perception that farmers are mainly focused on the economic aspect of farming and less on animal welfare, the perception that animals do not have a good quality of life while housed on farms, and the perception that animals are not adequately transported and slaughtered, negatively impact on perceptions about the general conditions of animal welfare in the poultry, beef, and dairy supply chains.

## Introduction

In the last decades, there is the increasing public concern about the welfare of animals used for food production, with citizens, particularly from developed countries, questioning the intensification of animal production systems and requiring that farm animals have a good life [1]. In New Zealand, the United States, and European Union countries, pressure from society has led to changes in animal production systems, which resulted in the improvements of animal welfare standards [1–3]. However, in some cases, stakeholders (i.e. farmers, veterinarians, industry participants, scientists, citizens, and consumers) have different views and concerns about animal welfare, which can hamper the implementation of practices that improve animal welfare standards [4–5]. Therefore, if we want to successfully implement strategies to improve the animal welfare standards, it is important to understand the concerns and perceptions of all stakeholders involved in the supply chains [1]. Particularly, important is the understanding of society’ perceptions about animal welfare because citizens play an important role in determining what is acceptable or not when it comes to the conditions of animal welfare in supply chains. For instance, citizens can pressure the government to implement laws to improve the welfare of animals used for food production, or they can choose to buy a certain type of products that guarantee good animal welfare standards [6].

Much of the research to date about citizens’ views, attitudes, and perceptions about animal welfare has been conducted on countries in North America [4, 7, 8], Europe [9–16] and in China [17]. In Brazil, one of the leading countries in livestock production [18], studies on citizens’ perceptions about animal welfare are emerging, but there is a need to deepen our understanding of how Brazilian society perceives animal welfare conditions. Previous research conducted in Brazil has focused on citizens’ perceptions of specific dairy farming practices, such as contentious practices [3], zero-grazing, and calf-separation [19]. In another study conducted in Brazil, Yunes et al. [20] investigated, specifically, citizens’ opinions and attitudes about farm animal production systems. Instead of focusing on a specific farming practice or in farm animal production systems, our study moves beyond the previous literature by investigating the perceptions of Brazilian citizens about the general conditions of animal welfare on poultry, beef, and dairy supply chains and the factors that explain their perceptions. We analyzed whether socio-demographic characteristics, awareness about animal welfare, knowledge about supply chains, perceptions about farming, perceptions about the quality of life of farm animals, perceptions about the use of animals for human consumption, and perceptions about the conditions of transport and slaughtering in each supply chain would have an impact on a measure of citizens’ perceptions about the general conditions of animal welfare in each of the three supply chains. By documenting the relationship between perceptions of general conditions of animal welfare and explanatory factors, our study contributes to the development of explanatory models for the social formation of perceptions about animal welfare conditions.

In light of the foregoing, the aim of this study was to understand the perceptions of Brazilian citizens about the general conditions of animal welfare in the poultry, beef, and dairy supply chains. These three supply chains play an important role in the Brazilian economy [18], but little is known about Brazilian citizens’ perceptions of the general conditions of animal welfare on these three supply chains. A failure to engage with citizens’ concerns about animal welfare conditions might risk the long-term sustainability of food industries [20].

## Material and methods

### Survey and sampling

We made three similar questionnaires: one for poultry supply chain, one for beef supply chain, and one for the dairy supply chain. The questionnaires consisted of three groups of questions and were adapted from Boogaard et al. [13] and Kupsala et al. [14]. In the first group of questions, we measured participants’ socio-demographic characteristics. In the second group of questions, we measured participants’ perceptions about the general conditions of animal welfare on each supply chain and other questions related to animal welfare. All the variables and scales used are presented in S1 Table. In the third group of questions, we used statements to measure participants’ perceptions about animal welfare. Statements used to measure participants’ perceptions are presented in S2 Table. All questions and statements were specifically adapted for each of the three supply chains questionnaires. This project received research ethics board approval from Federal University of Grande Dourados/Faculty of Management, Accounting and Economics. Before starting data collection, the questionnaire was tested with 20 participants. No substantial changes were necessary. All the questions were translated to Portuguese. Survey questions in the original language are presented in S1 Survey. To ensure correct translation, the questions were translated back to English. Survey questions in English are presented in S2 Survey.

To collect the data, we conducted an anonymous online survey. In the first step, we contacted, by phone, the human resource departments in several universities across Brazil. We used purposive sampling to select the universities. In our first contact with the universities, we explained the purpose of our research and asked if the department would forward a survey link to the personal e-mail of students, professors, and administration staff. Upon acceptance, we sent a follow-up e-mail to human resource departments with the survey link and a brief description of the research, which was then disseminated through e-mail for the academic community. Eighteen universities disseminated the questionnaire (6 universities for each of the three supply chains). We received 1614 questionnaires of which three were disregarded because they were incomplete. The final number of questionnaires was 728 for the poultry supply chain, 586 for the beef supply chain, and 300 for the dairy supply chain. The data collection took place from November 2016 until December 2017.

The selection of participants was restricted only to academic community members, and therefore our results must be viewed with care because our sample was more educated than the Brazilian population. The higher level of education is positively correlated with awareness and concerns about animal welfare issues [21], and therefore it is likely that participants in our sample held more progressive views toward animal welfare than a representative sample of the Brazilian population.

### Statistical analysis

Statistical analysis was conducted in three steps. In the first step, we used descriptive statistics to characterize the sample and to describe the main features of the measures. In the second step, we used factor analysis to reduce the number of items used to represent participants’ perceptions about animal welfare. The principal component was used as the extraction method. The criterion to define the number of factors was an eigenvalue greater than one [22]. Items were included in a factor when they presented factor loadings greater than 0.5. Factors scores were generated for subsequent analysis [22].

In the third step, we ran three logistic regression models. The three dependent variables were participants’ perceptions about the general conditions of animal welfare in each supply chain. In the original questionnaires, this variable was measured on a scale from 1 to 5 (S1 Table). In order to run the logistic models, we transformed the variable participants’ perceptions about the general conditions of animal welfare in each supply chain into a binary variable, where participants who answered 1 or 2 were gathered to a bad condition group (Bad:0) and participants who answered 3, 4 or 5 were gathered to a regular condition group (Regular:1). We acknowledge that dichotomizing this variable resulted in losing information. However, less than 15% of the participants answered 4 (good conditions) and 5 (very good conditions) for this question, and keeping the full scale would result in the complex analysis. We argue that such transformation brings more interpretability and simplicity to our analysis. In the logistic models, we tested whether socio-demographic characteristics, awareness about animal welfare, knowledge about supply chains, perceptions about farming, perceptions about the quality of life of farm animals, perceptions about the use of animals for human consumption, and perceptions about the conditions of transport and slaughtering would have an impact on a measure of citizens’ perceptions about the general conditions of animal welfare in each of the three supply chains. The significance level was p<0.05. Data is presented in S1 Raw Data.

## Results

### Descriptive statistics

Descriptive statistics of the variables used in the questionnaire are presented in Table 1. In the three questionnaires, participants’ socio-demographic characteristics were similar, except for age, education level, and income. In the three questionnaires, awareness about the animal welfare concept, level of knowledge about animal welfare regulations, and level of knowledge about the supply chains, were similar. Participants’ perceptions of the general conditions of animal welfare were similar for beef and dairy chain questionnaires, but somehow different for the poultry chain questionnaire.

**Table 1:** Descriptive statistics of the variables used in the questionnaires.

Variables	Poultry supply chain (n = 728)	Beef supply chain (n = 583)	Dairy supply chain (n = 300)
Conditions of animal welfare (%) (0:bad; 1:regular)–transformed variable	0:59.5; 1:40.5	0:41.12; 1:58.87	0:41.0; 1:59.0
Conditions of animal welfare (%) (1:very bad; 2:bad; 3:regular; 4:good; 5:very good)–original variable	1:29.7; 2:29.9; 3:30.63; 4:9.06; 5: 1.23	1:14.0; 2:27.1; 3:42.83; 4:14.85; 5:1,19	1:14.0; 2:27.0; 3:47.3; 4:10.3; 5:1.3
Age (years) (mean and standard deviation in brackets)	28 (10)	29 (10)	33 (13)
Gender (%) (0:female; 1:male)	0:65.1; 1: 34.9	0:56.7; 1:43.3	0:61.3; 1:38.7
Education (%) (1:incomplete elementary school; 2:complete elementary school; 3:incomplete high school; 4:complete high school; 5:incomplete bachelor degree; 6:complete bachelor degree; 7:incomplete postgraduate studies; 8:complete postgraduate studies)	1:0.0; 2:0.4; 3:0.7; 4:4.5; 5:49.9; 6:9.1; 7:11.7; 8:23.8	1:0.0; 2:0.2; 3:0.9; 4:5.6; 5:42.2; 6:8.4; 7:10.9; 8:31.9	1:0.0; 2:0.0; 3:0.0; 4:1.7; 5:32.0; 6:6.0; 7:14.0; 8:46.3
Field of study (%) (0:not related to agricultural/veterinary sciences; 1:related to agricultural/veterinary sciences)	0:70.2; 1:29.8	0:74.2; 1:25.8	0:75.7; 1:24.3
Pet ownership (%) (0:no; 1:yes)	0:23.6; 1:76.4	0:30.5; 1:69.5	0:36.3; 1:63.7
Income (%) (1:less than R$2.500,00; 2:R$2.500,00-R$5.000,00; 3:R$5.000,00-R$10.000,00; 4:more than R$10.000,00)	1:59.1; 2:18.3; 3:13.7; 4:8.9	1:49.7; 2:21.5; 3:19.5; 4:9.4	1:37.0; 2:18.3 3:26.3; 4:18.3
Contact with farm animals (%) (0:no; 1:yes)	0:42.6; 1:57.4	0:30.0; 1:70.0	0:26.3; 1:73.7
Local of residence (%) (1:urban; 2:rural; 3:both)	1:87.0; 2:2.7; 3:10.3	1:86.0; 2:4.4; 3:9.6	1:91.3; 2:1.7; 3:7.0
Consumption of animal products (Number of times) (mean; and standard deviation in brackets)	3 (2)	4 (2)	5 (4)
Awareness about animal welfare (%) (0:no; 1:yes)	0:15.7; 1: 84.3	0:18.3; 1:81.7	0:19.7; 1:80.3
Knowledge about the supply chain (%) (0:None; 1:I kind of know it; 2:I know it very well)	0:21.7; 1: 66.9; 2:11.4	0:24.1; 1:61.8; 2:14.2	0:32.3; 1:56.7; 2:11.0
Knowledge about the animal welfare regulations (%) (0:None; 1:I kind of know it; 2:I know it very well)	0:38.2; 1:50.3; 2:11.5	0:44.7; 1:44.9; 2:10.4	0:51.7; 1:37.3; 2:11.0
Comparison among national and international farm animal production (%) (1:strongly disagree; 2:disagree; 3:neutral; 4:agree; 5:strongly agree)	1:17.3; 2:18.7; 3:57.0; 4:5.6; 5:1.4	1:14.7; 2:23.0; 3:49.5; 4:11.3; 5:1.5	1:17.7; 2:20.0; 3:52.7; 4:9.0; 5:0.7
Transportation^a^ (%) (1:strongly disagree; 2:disagree; 3:neutral; 4:agree; 5:strongly agree)	1:40.4; 2:37.4; 3:8.8; 4:7.7; 5:5.8	1:27.6; 2:42.0; 3:13.9; 4:13.5; 5:2.91	-
Slaughtering^a^ (%) (1:strongly disagree; 2:disagree; 3:neutral; 4:agree; 5:strongly agree)	1:32.7; 2:36.3; 3:12.1; 4:11.4; 5:7.6	1:25.7; 2:41.1; 3:10.2; 4:16.3; 5:5.7	-

^a^ We have not measured transportation and slaughtering for the dairy supply chain.

### Factor analysis

Results of the factor analysis are presented in S3 Table. For the three supply chains, there were three factors with eigenvalue above 1.0. These three factors explained 67.5%, 62.1%, and 63.7% of the total variance in the poultry, beef, and dairy supply chains, respectively. Results of the factors loadings were also similar in the three supply chains. Following Boogaard et al. [13], we named the first factor as Farmers’ Image (FI), the second factor as Life Quality of Farm Animals (LQFA), and the third factor as Use of Animals for Human Consumption (UAHC). The first factor describes participants’ perceptions about farmers. The items of this factor were negatively formulated in the questionnaire, so the higher the participants scored on FI, the more they agreed that farmers are mainly focused on the economic aspect of farming and less on the animal welfare. The second factor describes participants’ perceptions of the conditions of animal welfare in farming. The higher the participants scored on LQFA, the more they agreed that animals have a good quality of life while housed on farms. The third factor describes participants’ perceptions about the use of animals for human consumption. The lower the participants scored on UAHC, the more they agreed that humans are allowed to use animals for consumption.

Descriptive statistics about the statements used to measure participants’ perceptions about animal welfare are presented in S2 Table. For the statements related to FI (Perc_1_, Perc_2_, Perc_3_, Perc_4_), the mean was above or close to 4, which indicates that participants agreed that most farmers focus too much on the economic aspect of farming and less on the animal welfare. For the statements related to LQFA (Perc_5_, Perc_6_, Perc_7_, Perc_8_), the mean was below or close to 3, which indicates that participants did not agree that animals have a good quality of life while housed on farms. For the statements related to UAHC (Perc_9_, Perc_10_), the mean was below or close to 2, which indicates that participants agreed that humans are allowed to use animals for consumption.

### Logistic regression models

We tested whether socio-demographic characteristics, awareness about animal welfare, knowledge about supply chains, perceptions about farming, perceptions about the quality of life of farm animals, perceptions about the use of animals for human consumption, and perceptions about the conditions of transport and slaughtering would have an impact on a measure of citizens’ perceptions about the general conditions of animal welfare in each of the three supply chains. Results of the three logistic regression models are present in Table 2.

**Table 2:** Logistic regression models of the Brazilian citizen perceptions about the conditions of animal welfare on poultry, beef, and dairy supply chains.

Independent variables	Conditions of animal welfare in poultry supply chain			Conditions of animal welfare in beef supply chain			Conditions of animal welfare in dairy supply chain		
	B	S.E.	Exp (B)	B	S.E.	Exp (B)	B	S.E.	Exp (B)
Age	0.016	0.011	1.016	-0.010	0.011	0.990	0.001	0.013	1.001
Gender	-0.178	0.211	0.837	0.081	0.222	1.084	-0.357	0.339	0.700
Pet ownership	0.245	0.235	1.278	-0.026	0.230	0.974	-0.093	0.345	0.911
Field of study	-0.885*	0.254	0.413	-0.253	0.295	0.776	-1.596*	0.510	0.203
Contact with farm animals	-0.376*	0.228	0.686	-0.124	0.245	0.833	0.150	0.383	1.162
Consumption of animal products	0.027	0.046	1.028	0.065	0.044	1.067	0.047	0.046	1.048
Awareness about animal welfare	0.208	0.283	1.231	0.141	0.283	1.152	0.323	0.417	1.381
Knowledge about the supply chain	-0.551*	0.218	0.576	0.077	0.229	1.080	-0.719*	0.334	0.487
Knowledge about the animal welfare regulations	0.059	0.192	1.061	0.010	0.204	1.010	-0.115	0.307	0.891
Comparison among national and international farm animal production	0.156	0.122	1.169	0.075	0.204	1.078	-0.083	0.180	0.921
Transportation^a^	0.101	0.110	1.106	0.351*	0.140	1.421	-	-	-
Slaughtering^a^	0.318*	0.109	1.375	0.301*	0.133	1.351	-	-	-
Farmers’ Image (FI)	-0.872*	0.116	0.418	-0.729*	0.122	0.483	-0.669*	0.179	0.512
Life quality of farm animals (LQFA)	0.797*	0.116	2.219	0.742*	0.131	2.100	1.535*	0.215	4.642
Use of animals for human consumption (UAHC)	-0.829*	0.141	0.437	-0.745*	0.132	0.475	-0.498*	0.184	0.608
Constant	-1236	0.656	0.291	-1.068	0.699	0.344	2.552*	0.972	12.834
Likelihood logarithm	645.370			557.895			265.813		
Chi-square value	337.534			235.918			140.303		

^a^We have not measured transportation and slaughtering for the dairy supply chain.

*p <0.05.

The socio-demographic characteristics, such as age, gender, pet ownership, and consumption of animal products, did not significantly have an impact on participants’ perceptions about the general conditions of animal welfare in any supply chain. Participants who reported the previous contact with poultry farms were more likely to perceive the general conditions of animal welfare in the poultry supply chain as being bad compared to participants who had not reported the previous contact. Participants in the fields of study related to agricultural/veterinary sciences were more likely to perceive the general conditions of animal welfare in the poultry and dairy supply chains as being bad compared to participants out of these fields. Participants who reported a higher level of knowledge about poultry and dairy supply chains were more likely to perceive the general conditions of animal welfare in the poultry and dairy supply chains as being bad compared to those participants who reported a lower level of knowledge about these supply chains. Participants who perceived that animals are adequately transported and slaughtered were more likely to perceive the general conditions of animal welfare in the poultry and beef supply chains as being regular compared to those participants who perceived that animals are not adequately transported and slaughtered. Participants who perceived that farmers are mainly focused on the economic aspect of farming and less on the animal welfare (FI) were more likely to perceive the general conditions of animal welfare in the poultry, beef, and dairy supply chains as being bad compared to those who perceived that farmers are more focused on animal welfare and less onn the economic aspect of farming. Moreover, participants who perceived that animals have a good quality of life while housed on farms (LQFA) were more likely to perceive the general conditions of animal welfare in the poultry, beef, and dairy supply chains as being regular compared to those who perceived that animals do not have a good quality of life while housed on farms. Finally, participants who perceived that humans are allowed to use animals for consumption (UAHC) were more likely to perceive the general conditions of animal welfare in the poultry, beef, and dairy supply chains as being regular compared to those who perceived that humans are not allowed to use animals for consumption.

## Discussion and concluding comments

The vast majority of participants perceived the general conditions of animal welfare in the poultry, beef, and dairy supply chains as very bad, bad, or regular. In their review, Clark et al. [21] showed that citizens from developing and developed countries have more negative than positive perceptions about the animal welfare conditions, which is in line with our findings.

Results of the three logistic regression models were similar. Most socio-demographic characteristics did not have an impact on perceptions about the general conditions of animal welfare in any of the three supply chains. In contrast, Kupsala et al. [14] found that women, younger people, and people who are pet owners perceived the conditions of animal welfare in Finland more negatively than men, older people, and people who are not pet owners. These contradictory results might be explained, while Kupsala et al. [14] focused on general public, our sample is restricted to the academic community, which yields specific results that cannot be generalized to the Brazilian population. We recommend that future research should focus on the Brazilian general public to investigate the role of socio-demographic characteristics in shaping perceptions about animal welfare conditions.

In our logistic regression models, we had three variables related to participants’ knowledge about the supply chains: their background in agricultural/veterinary sciences, a self-reported level of knowledge, and previous contact with farms. Results of the logistic regression models showed that these variables related to knowledge about the supply chains had a negative impact on perceptions about the general conditions of animal welfare in the poultry and dairy chains. These results can be explained by a growing body of literature indicating that as more people know about farming practices, the more they think that these practices do not provide a good quality of life for farm animals [7, 12, 19]. In contrast, the results of the logistic regression showed that variables related to knowledge about the supply chain and farming did not have an impact on perceptions about the general conditions of animal welfare in the beef chain. These results might be explained by the difference in animal production systems used in the three supply chains in Brazil. The predominant production systems in poultry and dairy supply chains in Brazil are intensive, where animals live mostly confined [1, 19] whereas, in the beef supply chain, animals are reared in more extensive systems [18]. Intensive production systems are usually perceived by citizens as unnatural and by providing less animal welfare compared to extensive systems [21]. Therefore, in our sample, participants who had more knowledge about animal production systems might know that poultry and dairy cows are mostly housed in confined housing systems and therefore, were more likely to perceive the conditions of animal welfare in poultry and dairy supply chains as being bad. In contrast, participants who have more knowledge about animal production systems might know that beef cattle are mostly reared in extensive production systems, and therefore knowledge did not have an impact on their perceptions about the general conditions of animal welfare in beef chain. These results suggest that increasing citizens’ education about animal production systems and practices used in supply chains will decrease their acceptance of such production systems and practices, particularly in supply chains with more intensive production systems. Ventura et al. [23] also claimed that education and exposure to livestock farming might not improve citizens’ perceptions that farm animals have a good life.

Results of the logistic regressions also showed that perceptions that farmers are mainly focused on the economic aspect of farming, perceptions that animals do not have a good quality of life in farms, and perceptions that animals are not adequately transported and slaughtered, had a negative impact on perceptions about the general conditions of animal welfare. These results indicate that perceptions about animal welfare conditions on farming, transportation, and slaughtering shape the perceptions about the general conditions of animal welfare.

A potential limitation of this study is the participants selected only from the academic community. In comparison to the Brazilian population, our sample is younger, more educated, and earns a higher income [24]. Although we acknowledge that our sample is unbalanced in terms of education, income, and age, we argue that academic community members have more access to information that might drive changes in production systems. The aforementioned limitation is even more challenging because of the non-probabilistic characteristic of the purposive sampling method used to select universities. We acknowledge that a probabilistic sampling method to select universities would produce less biased results, and therefore the purposive sampling method is a shortcoming of this research. Because of a technical issue in the survey link sent to participants, transportation and slaughtering questions were not measured for the dairy supply chain, which resulted in another limitation of this study.

## Supporting information

S1 Table Variables, questions, and scales used in the questionnaire. (DOCX)

S2 Table Descriptive statistics of the statements used to measure participants’ perceptions about animal welfare. (DOCX)

S3 Table Factor loading matrix for the perceptions items for each chain, with factor loadings greater than |0.5| in bold. (DOCX)

S1 Survey Survey questions in the original language (Portuguese). (DOCX)

S2 Survey Survey questions in English. (DOCX)

S1 Raw Data (XLS)
