# Supplementary material for: How do Brazilian citizens perceive animal welfare conditions in poultry, beef, and dairy supply chains?
Source: PLoS One. 2018 Dec 19;13(12):e0202062. doi: 10.1371/journal.pone.0202062 (PMC6300285; doi:10.1371/journal.pone.0202062)
Supplement: S1 Table — (DOCX) [file pone.0202062.s001.docx]

**S1 Table. Variables, questions and scales used in the questionnaire**

| Variables | Questions | Scales |
| --- | --- | --- |
| Opinion about the conditions of animal welfare in the supply chain | In your opinion, the conditions of animal welfare in the poultry ^a^ supply chain are: | 1:very bad; 2: bad; 3:regular; 4:good; 5:very good |
| Age | How old are you? | years |
| Gender | Gender | 0:female; 1:male |
| Education | What is your education level? | 1:incomplete elementary school;  2:complete elementary school;  3:incomplete high school;  4:complete high school;  5:incomplete bachelor degree;  6:complete bachelor degree;  7:incomplete postgraduate studies;  8:complete postgraduate studies |
| Field of study | Is your field of study related to agricultural or veterinary sciences? | 0:no; 1:yes |
| Pet ownership | Are you a pet owner? | 0:no; 1:yes |
| Income | What is your monthly income ^b^? | 1:less than R$2.500,00; 2:R$2.500,00 – R$5.000,00; 3:R$5.000,00 – R$10.000,00; 4: more than R$10.000,00 |
| Contact with farm animals | Have you ever had contact with poultry ^a^ farms? | 0:no; 1:yes |
| Local of residence | Do you live in urban or rural area? | 1: urban; 2:rural; 3:both |
| Consumption of animal products | How often do you eat chicken ^a^ (per week)? | Number of times |
| Awareness about animal welfare | Have you ever heard about animal welfare? | 0:no; 1:yes |
| Knowledge about the supply chain | What is your level of knowledge about the poultry ^a^ supply chain? | 0: None; 1: I kind of know it; 2: I know it very well |
| Knowledge about the animal welfare regulations | What is your level of knowledge about animal welfare regulations? | 0: None; 1: I kind of know it; 2: I know it very well |
| Comparison among national and international farm animal production | In your opinion, poultry ^a^ farmers in Brazil provide more animal welfare conditions to their animals compared to American and European farmers: | 1:strongly disagree; 2:disagree; 3:neural; 4:agree; 5:strongly agree |
| Transportation ^c^ | Chickens ^a^ are transported adequately to slaughterhouse | 1:strongly disagree; 2:disagree; 3:neural; 4:agree; 5:strongly agree |
| Slaughtering ^c^ | Chickens ^a^ are adequately slaughtered | 1:strongly disagree; 2:disagree; 3:neural; 4:agree; 5:strongly agree |

^a^ The words ‘poultry or chicken’ was replaced by the word ‘beef or cattle’ in the beef questionnaire, and by the words ‘dairy or milk’ in the dairy questionnaire.

^b^ Measured in Reais (R$) - R$1000,00= ± $310,00.

^c^ We have not measured transportation and slaughtering for the dairy supply chain
