# Supplementary material for: How do Brazilian citizens perceive animal welfare conditions in poultry, beef, and dairy supply chains?
Source: PLoS One. 2018 Dec 19;13(12):e0202062. doi: 10.1371/journal.pone.0202062 (PMC6300285; doi:10.1371/journal.pone.0202062)
Supplement: S2 Table — (DOCX) [file pone.0202062.s002.docx]

**S2 Table. Descriptive statistics of the statements used to measure participants’ perceptions about animal welfare**

| Items | Statements ^a b^ | Poultry supply chain (n=728)  Mean (SD) | Beef supply chain (n=583)  Mean (SD) | Dairy supply chain (n=300)  Mean (SD) |
| --- | --- | --- | --- | --- |
| Perc_1_ | Most poultry ^c^ farmers focus too much on management instead of their animals | 4.23 (1.00) | 3.66 (1.16) | 3.71 (1.17) |
| Perc_2_ | Most poultry ^c^ farmers consider their animals too much as means of production | 4.50 (0.84) | 4.28 (1.00) | 4.20 (0.96) |
| Perc_3_ | Most poultry ^c^ farmers solely treat animals properly as long as it is financially beneficial | 4.4 (0.92) | 4.25 (0.99) | 4.22 (1.03) |
| Perc_4_ | Most poultry ^c^ farmers economize on animal welfare | 4.08 (1.10) | 3.82 (1.13) | 3.96 (1.03) |
| Perc_5_ | Chickens ^c^ on farms have quite a good life | 2.23 (1.18) | 2.45 (1.07) | 2.26 (0.98) |
| Perc_6_ | Most poultry ^c^ farmers give their animals a comfortable life | 2.14 (1.16) | 2.42 (1.12) | 2.29 (1.09) |
| Perc_7_ | Most poultry ^c^ farmers care about their animals | 2.20 (1.21) | 2.67 (1.22) | 2.82 (1.24) |
| Perc_8_ | Most poultry ^c^ farmers are in daily contact with their animals | 2.96 (1.25) | 2.74 (1.20) | 3.15 (1.28) |
| Perc_9_ | Humans are allowed to use chicken ^c^ for consumption ^d^ | 1.77 (1.17) | 1.54 (0.91) | 1.79 (1.11) |
| Perc_10_ | I feel guilty when I eat chicken ^c^ | 2.43 (1.52) | 2.12 (1.42) | 1.92 (1.32) |

^a^ All the statements were preceded by ‘What is your level of agreement for each statement below’

^b^ All the statements were measured using a Likert-type scale (1:strongly disagree; 2:disagree; 3:neutral; 4:agree; 5:strongly agree).

^c^ The words ‘poultry or chicken’ was replaced by the word ‘beef’ in the beef questionnaire, and by the words ‘dairy or milk’ in the dairy questionnaire.

^d^ Variable recoded, as it was negatively formulated in the questionnaire.
