# Supplementary material for: How do Brazilian citizens perceive animal welfare conditions in poultry, beef, and dairy supply chains?
Source: PLoS One. 2018 Dec 19;13(12):e0202062. doi: 10.1371/journal.pone.0202062 (PMC6300285; doi:10.1371/journal.pone.0202062)
Supplement: S3 Table — (DOCX) [file pone.0202062.s003.docx]

**S3 Table. Factor loading matrix for the perceptions items for each chain, with factor loadings greater than |0.5| in bold**

| Items | Poultry supply chain | | | Beef supply chain | | | Dairy supply chain | | |
| --- | --- | --- | --- | --- | --- | --- | --- | --- | --- |
|  | Factors | | | Factors | | | Factors | | |
|  | 1^a^ | 2^b^ | 3^c^ | 1^a^ | 2^b^ | 3^c^ | 1^a^ | 2^b^ | 3^c^ |
| Perc_1_ | **0.741** | -0.146 | 0.117 | **0,708** | -0,114 | 0,067 | **0.683** | -0.142 | 0.103 |
| Perc_2_ | **0.831** | -0.138 | 0.039 | **0,809** | -0,092 | 0,034 | **0.767** | -0.214 | 0.087 |
| Perc_3_ | **0.814** | -0.055 | 0.026 | **0,775** | -0,118 | 0,046 | **0.783** | -0.093 | 0.062 |
| Perc_4_ | **0,751** | -0,255 | 0,222 | **0,738** | -0,270 | 0,131 | **0.732** | -0.363 | 0.032 |
| Perc_5_ | -0.128 | **0.816** | -0.085 | -0,129 | **0,768** | -0,147 | -0.129 | **0.834** | -0.154 |
| Perc_6_ | -0.144 | **0.871** | -0.028 | -0,169 | **0,842** | -0,128 | -0.209 | **0.848** | -0.044 |
| Perc_7_ | -0.271 | **0.750** | -0.131 | -0,111 | **0,744** | -0,152 | -0.234 | **0.746** | -0.139 |
| Perc_8_ | -0.050 | **0.655** | -0.211 | -0,121 | **0,534** | 0,061 | * | * | * |
| Perc_9_ | 0.109 | -0.116 | **0.860** | 0,039 | -0,087 | **0,863** | 0.061 | -0.125 | **0.841** |
| Perc_10_ | 0.132 | -0.183 | **0.835** | 0,147 | -0,131 | **0,843** | 0.097 | -0.160 | **0.821** |
| Variance explained (%) | 38.81 | 16.38 | 12.35 | 34.30 | 15.25 | 12.55 | 39.11 | 13.67 | 10.90 |
| Eingenvalue | 3.881 | 1.639 | 1.231 | 3.41 | 1.525 | 1.255 | 3.911 | 1.367 | 1.090 |

^a^ Farmers’ Image (FI)

^b^ Life Quality of Farm Animals (LQFA)

^c^ Use of Animals for Human Consumption (UAHC)

* All factors loadings for this item were below the threshold value of 0.5. Therefore, this item was excluded in the factor analysis of dairy supply chain.
