# Supplementary material for: How do Brazilian citizens perceive animal welfare conditions in poultry, beef, and dairy supply chains?
Source: PLoS One. 2018 Dec 19;13(12):e0202062. doi: 10.1371/journal.pone.0202062 (PMC6300285; doi:10.1371/journal.pone.0202062)
Supplement: S1 Survey — (DOCX) [file pone.0202062.s004.docx]

**S4 Survey. Survey questions in the original language (Portuguese).**

**Questionário referente à cadeia produtiva da carne de frango**

**Características socioeconômicas:**

1) Quantos anos você tem?

( ) anos

2) Qual seu nível educacional?

( ) Ensino fundamental incompleto

( ) Ensino fundamental completo

( ) Ensino médio incompleto

( ) Ensino médio completo

( ) Graduação incompleta

( ) Graduação completa

( ) Pós-graduação incompleta

( ) Pós-graduação completa

3) Você tem algum animal de estimação?

( ) Sim

( ) Não

4) Qual a sua renda mensal?

( ) Até R$ 2.500,00

( ) De R$ 2.500,00 a 5.000,00

( ) De R$ 5.000,00 a 10.000,00

( ) Acima de R$ 10.000,00

5) Sexo:

( ) Masculino

( ) Feminino

( ) Outro

6) Você tem/teve contato com frangos de corte (aves destinadas à produção de carne) durante sua vida?

( ) Sim

( ) Não

7) Se sim, qual o tipo de contato?

( ) Morei em uma propriedade rural que produzia frangos de corte

( ) Alguém da família tinha ou tem propriedade rural que produz frangos de corte

( ) Visitei propriedades rurais que produziam frango de corte, mas nunca tive contato direto com esses animais

( ) Trabalho ou trabalhei na área de avicultura

( ) Outro

8) Qual a sua profissão?

.....................................................................

9) Se você for estudante, qual curso está realizando?

......................................................................

10) Qual a cidade e estado onde você reside:

Cidade: ..........................................................................

Estado: ........................................................................

11) Você vive no meio urbano ou meio rural?

( ) Meio urbano

( ) Meio rural

( ) Ambos

12) Em média, quantas vezes por semana você consome carne de frango?

...............

13) Você já ouviu falar sobre bem-estar animal?

( ) Não

( ) Sim

14) Em uma escala de 1 a 5, na sua opinião, as condições atuais de bem-estar dos frangos na cadeia produtiva avícola são:

Muito ruins 1 2 3 4 5 Muito boas

15) Em uma escala de 1 a 5, qual é seu nível de preocupação com o bem-estar dos frangos na cadeia produtiva avícola

Muito baixo 1 2 3 4 5 Muito alto

16) Com relação às suas percepções sobre bem-estar de frangos de corte, qual seu grau de concordância para cada uma das sentenças abaixo?

Discordo totalmente 1 2 3 4 5 Concordo totalmente Não sei

A maioria dos produtores rurais de frango de corte se preocupa mais com o gerenciamento da granja que com as aves

A maioria dos produtores rurais de frango de corte considera suas aves somente como uma fonte de renda

Para a maioria dos produtores rurais de frango de corte, o tratamento adequado dado às aves é possível somente se isso significar maior lucro

A maioria dos produtores rurais de frango de corte tenta economizar e não garante o bem-estar das aves

A vida humana é mais importante que a vida de um frango

Nas propriedades rurais, os frangos têm uma vida muito boa

A maioria dos produtores rurais proporciona aos frangos uma vida confortável

A maioria dos produtores rurais de frango de corte se importa muito com suas aves

A maioria dos produtores rurais de frango de corte tem contato diário com suas aves

Humanos podem consumir carne de frango

Eu me sinto culpado quando eu consumo carne de frango

Os frangos são transportados para os abatedouros de maneira adequada

Os frangos são abatidos de maneira adequada

17) Na sua opinião, os produtores de frango de corte do Brasil cumprem melhor as necessidades de bem-estar das aves que os produtores dos Estados Unidos e União Europeia?

Discordo totalmente 1 2 3 4 5 Concordo totalmente

18) Qual o seu nível de conhecimento sobre a criação de frangos de corte?

( ) Nenhum

( ) Conheço razoavelmente

( ) Conheço bem

19) Qual o seu nível de conhecimento sobre leis que asseguram o bem-estar dos animais?

( ) Nenhum

( ) Conheço razoavelmente

( ) Conheço bem

**Questionário referente à cadeia produtiva da carne bovina**

**Características socioeconômicas:**

1) Quantos anos você tem?

( ) anos

2) Qual seu nível educacional?

( ) Ensino fundamental incompleto

( ) Ensino fundamental completo

( ) Ensino médio incompleto

( ) Ensino médio completo

( ) Graduação incompleta

( ) Graduação completa

( ) Pós-graduação incompleta

( ) Pós-graduação completa

3) Você tem algum animal de estimação?

( ) Sim

( ) Não

4) Qual a sua renda mensal?

( ) Até R$ 2.500,00

( ) De R$ 2.500,00 a 5.000,00

( ) De R$ 5.000,00 a 10.000,00

( ) Acima de R$ 10.000,00

5) Sexo:

( ) Masculino

( ) Feminino

( ) Outro

6) Você tem/teve contato com bovinos destinados à produção de carne durante sua vida?

( ) Sim

( ) Não

7) Se sim, qual o tipo de contato?

( ) Morei em uma propriedade rural que produzia bovinos de corte

( ) Alguém da família tinha ou tem propriedade rural que produz bovinos de corte

( ) Visitei propriedades rurais que produziam bovinos de corte, mas nunca tive contato direto com esses animais

( ) Trabalho ou trabalhei na área da bovinocultura de corte

( ) Outro

8) Qual a sua profissão?

.....................................................................

9) Se você for estudante, qual curso está realizando?

......................................................................

10) Qual a cidade e estado onde você reside:

Cidade: ..........................................................................

Estado: ........................................................................

11) Você vive no meio urbano ou meio rural?

( ) Meio urbano

( ) Meio rural

( ) Ambos

12) Em média, quantas vezes por semana você consome carne bovina?

...............

13) Você já ouviu falar sobre bem-estar animal?

( ) Não

( ) Sim

14) Em uma escala de 1 a 5, na sua opinião, as condições atuais de bem-estar dos animais na cadeia produtiva da carne bovina são:

Muito ruins 1 2 3 4 5 Muito boas

15) Em uma escala de 1 a 5, qual é seu nível de preocupação com o bem-estar dos animais na cadeia produtiva da carne bovina?

Muito baixo 1 2 3 4 5 Muito alto

16) Com relação às suas percepções sobre bem-estar de bovinos de corte, qual seu grau de concordância para cada uma das sentenças abaixo?

Discordo totalmente 1 2 3 4 5 Concordo totalmente Não sei

A maioria dos produtores rurais de carne bovina se preocupa mais com o gerenciamento da propriedade que com o rebanho bovino

A maioria dos produtores rurais de carne bovina considera seu rebanho bovino somente como uma fonte de renda

Para a maioria dos produtores rurais de carne bovina, o tratamento adequado dado aos bovinos é possível somente se isso significar maior lucro

A maioria dos produtores rurais de carne bovina tenta economizar e não garante o bem-estar dos bovinos

A vida humana é mais importante que a vida de um bovino

Nas propriedades rurais, os bovinos têm uma vida muito boa

A maioria dos produtores rurais proporciona aos bovinos uma vida confortável

A maioria dos produtores rurais de carne bovina se importa muito com seu rebanho bovino

A maioria dos produtores rurais de carne bovina tem contato diário com seu rebanho bovino

Humanos podem consumir carne bovina

Eu me sinto culpado quando eu consumo carne bovina

Os bovinos são transportados para os frigoríficos de maneira adequada

Os bovinos são abatidos de maneira adequada

17) Na sua opinião, os produtores de bovinos de corte do Brasil cumprem melhor as necessidades de bem-estar dos bovinos que os produtores dos Estados Unidos e União Europeia?

Discordo totalmente 1 2 3 4 5 Concordo totalmente

18) Qual o seu nível de conhecimento sobre a criação de bovinos para a produção de carne?

( ) Nenhum

( ) Conheço razoavelmente

( ) Conheço bem

19) Qual o seu nível de conhecimento sobre leis que asseguram o bem-estar dos animais?

( ) Nenhum

( ) Conheço razoavelmente

( ) Conheço bem

**Questionário referente à cadeia produtiva bovina de leite**

**Características socioeconômicas:**

1. Quantos anos você tem?

( ) anos

2. Qual seu nível educacional?

( ) Ensino fundamental incompleto

( ) Ensino fundamental completo

( ) Ensino médio incompleto

( ) Ensino médio completo

( ) Graduação incompleta

( ) Graduação completa

( ) Pós-graduação incompleta

( ) Pós-graduação completa

3. Você tem algum animal de estimação?

( ) Sim

( ) Não

4. Qual a sua renda mensal?

( ) Até R$ 2.500,00

( ) De R$ 2.500,00 a 5.000,00

( ) De R$ 5.000,00 a 10.000,00

( ) Acima de R$ 10.000,00

5. Sexo:

( ) Masculino

( ) Feminino

( ) Outro

6. Você tem/teve contato com bovinos destinados à produção de leite durante sua vida?

( ) Sim

( ) Não

7. Se sim, qual o tipo de contato?

( ) Morei em uma propriedade rural que produzia frangos de corte

( ) Alguém da família tinha ou tem propriedade rural que produz frangos de corte

( ) Visitei propriedades rurais que produziam frangos de corte, mas nunca tive contato direto com esses animais

( ) Trabalho ou trabalhei na área de avicultura

( ) Outro

8. Qual a sua profissão?

.....

9. Se você for estudante, qual curso está realizando?

.....

10. Qual a cidade e estado onde você reside?

Cidade: ....

Estado: .....

11. Você vive no meio urbano ou meio rural?

( ) Meio urbano

( ) Meio rural

( ) Ambos

12. Em média, quantas vezes por semana você consome leite?

.....

13. Você já ouviu falar sobre bem-estar animal?

( ) Não

( ) Sim

14. Em uma escala de 1 a 5, na sua opinião, as condições atuais de bem-estar dos animais na cadeia da bovinocultura leiteira são:

Muito ruins 1 2 3 4 5 Muito boas

15. Em uma escala de 1 a 5, qual é seu nível de preocupação com o bem-estar dos animais na cadeia da bovinocultura de leite?

Muito baixo 1 2 3 4 5 Muito alto

16. Com relação às suas percepções sobre bem-estar de bovinos utilizados na produção de leite, qual seu grau de concordância para cada uma das sentenças abaixo?

Discordo totalmente 1 2 3 4 5 Concordo totalmente Não sei

A maioria dos produtores rurais de leite de vaca se preocupa mais com o gerenciamento da propriedade que com o rebanho

A maioria dos produtores rurais de leite de vaca considera seu rebanho bovino somente como uma fonte de renda

Para a maioria dos produtores rurais de leite de vaca, o tratamento adequado dado aos bovinos é possível somente se isso significar maior lucro

A maioria dos produtores rurais de leite de vaca tenta economizar e não garante o bem-estar dos bovinos

A vida humana é mais importante que a vida de um bovino

Nas propriedades rurais, os bovinos têm uma vida muito boa

A maioria dos produtores rurais de leite de vaca proporciona aos bovinos uma vida confortável

A maioria produtores rurais de leite de vaca se importa muito com seu rebanho bovino

A maioria dos produtores rurais de leite de vaca tem contato diário com seu rebanho bovino

Humanos podem consumir leite de vaca

Eu me sinto culpado quando eu consumo leite de vaca

17. Na sua opinião, os produtores rurais de leite do Brasil cumprem melhor as necessidades de bem-estar dos bovinos que os produtores dos Estados Unidos e União Europeia?

Discordo totalmente 1 2 3 4 5 Concordo totalmente

18. Qual o seu nível de conhecimento sobre a criação de bovinos para produção de leite?

( ) Nenhum

( ) Conheço razoavelmente

( ) Conheço bem

19. Qual o seu nível de conhecimento sobre leis que asseguram o bem-estar dos animais?

( ) Nenhum

( ) Conheço razoavelmente

( ) Conheço bem
