# Supplementary material for: How do Brazilian citizens perceive animal welfare conditions in poultry, beef, and dairy supply chains?
Source: PLoS One. 2018 Dec 19;13(12):e0202062. doi: 10.1371/journal.pone.0202062 (PMC6300285; doi:10.1371/journal.pone.0202062)
Supplement: S2 Survey — (DOCX) [file pone.0202062.s005.docx]

**S5 Survey. Survey questions in English.**

**Survey of poultry supply chain**

**Socioeconomic characteristics:**

1) How old are you?

( ) Years

2) What is your education level?

( ) incomplete elementary school;

( ) complete elementary school;

( ) incomplete high school;

( ) complete high school;

( ) incomplete bachelor degree;

( ) complete bachelor degree;

( ) incomplete postgraduate studies;

( ) complete postgraduate studies

3) Are you a pet owner?

( ) Yes

( ) No

4) What is your monthly income?

( ) less than R$2.500,00;

( ) R$2.500,00 – R$5.000,00;

( ) R$5.000,00 – R$10.000,00;

( ) more than R$10.000,00

5) Gender:

( ) Male

( ) Female

( ) Other

6) Have you ever had contact with poultry farms?

( ) Yes

( ) No

7) What’s your profession?

.....................................................................

8) If you are a student, what is your field of study?

......................................................................

9) Where do you live?

City: ..........................................................................

State: ........................................................................

10) Do you live in urban or rural area?

( ) Urban

( ) Rural

( ) Both

11) How often do you eat chicken (per week)??

...............

12) Have you ever heard about animal welfare?

( ) No

( ) Yes

13) In your opinion, the conditions of animal welfare in the poultry supply chain are:

Very bad 1 2 3 4 5 Very good

14) What is your level of concern about animal conditions in the poultry supply chain?

Very low 1 2 3 4 5 Very high

15) What is your level of agreement for each statement below’?

Strongly disagree Disagree Neutral Agree Strongly agree I do not know

1 2 3 4 5

Most poultry farmers focus too much on management instead of their animals

Most poultry farmers consider their animals too much as means of production

Most poultry farmers solely treat animals properly as long as it is financially beneficial

Most poultry farmers economize on animal welfare

Chickens on farms have quite a good life

Most poultry farmers give their animals a comfortable life

Most poultry farmers care about their animals

Most poultry farmers are in daily contact with their animals

Humans are allowed to use chicken for consumption

I feel guilty when I eat chicken

Chickens are transported adequately to slaughterhouse

Chickens are adequately slaughtered

16) In your opinion, poultry farmers in Brazil provide more animal welfare conditions to their animals compared to American and European farmers?

Strongly disagree 1 2 3 4 5 Strongly agree

17) What is your level of knowledge about the poultry supply chain?

( ) None;

( ) I kind of know it;

( ) I know it very well

18) What is your level of knowledge about animal welfare regulations?

( ) None;

( ) I kind of know it;

( ) I know it very well

**Survey of beef supply chain**

**Socioeconomic characteristics:**

1) How old are you?

( ) Years

2) What is your education level?

( ) incomplete elementary school;

( ) complete elementary school;

( ) incomplete high school;

( ) complete high school;

( ) incomplete bachelor degree;

( ) complete bachelor degree;

( ) incomplete postgraduate studies;

( ) complete postgraduate studies

3) Are you a pet owner?

( ) Yes

( ) No

4) What is your monthly income?

( ) less than R$2.500,00;

( ) R$2.500,00 – R$5.000,00;

( ) R$5.000,00 – R$10.000,00;

( ) more than R$10.000,00

5) Gender:

( ) Male

( ) Female

( ) Other

6) Have you ever had contact with beef farms?

( ) Yes

( ) No

7) What’s your profession?

.....................................................................

8) If you are a student, what is your field of study?

......................................................................

9) Where do you live?

City: ..........................................................................

State: ........................................................................

10) Do you live in urban or rural area?

( ) Urban

( ) Rural

( ) Both

11) How often do you eat beef (per week)??

...............

12) Have you ever heard about animal welfare?

( ) No

( ) Yes

13) In your opinion, the conditions of animal welfare in the beef supply chain are:

Very bad 1 2 3 4 5 Very good

14) What is your level of concern about animal conditions in the beef supply chain?

Very low 1 2 3 4 5 Very high

15) What is your level of agreement for each statement below’?

Strongly disagree Disagree Neutral Agree Strongly agree I do not know

1 2 3 4 5

Most beef farmers focus too much on management instead of their animals

Most beef farmers consider their animals too much as means of production

Most beef farmers solely treat animals properly as long as it is financially beneficial

Most beef farmers economize on animal welfare

Cattle on farms have quite a good life

Most beef farmers give their animals a comfortable life

Most beef farmers care about their animals

Most beef farmers are in daily contact with their animals

Humans are allowed to use cattle for consumption

I feel guilty when I eat beef

Cattle are transported adequately to slaughterhouse

Cattle are adequately slaughtered

16) In your opinion, beef farmers in Brazil provide more animal welfare conditions to their animals compared to American and European farmers?

Strongly disagree 1 2 3 4 5 Strongly agree

17) What is your level of knowledge about the beef supply chain?

( ) None;

( ) I kind of know it;

( ) I know it very well

18) What is your level of knowledge about animal welfare regulations?

( ) None;

( ) I kind of know it;

( ) I know it very well

**Survey of dairy supply chain**

**Socioeconomic characteristics:**

1) How old are you?

( ) Years

2) What is your education level?

( ) incomplete elementary school;

( ) complete elementary school;

( ) incomplete high school;

( ) complete high school;

( ) incomplete bachelor degree;

( ) complete bachelor degree;

( ) incomplete postgraduate studies;

( ) complete postgraduate studies

3) Are you a pet owner?

( ) Yes

( ) No

4) What is your monthly income?

( ) less than R$2.500,00;

( ) R$2.500,00 – R$5.000,00;

( ) R$5.000,00 – R$10.000,00;

( ) more than R$10.000,00

5) Gender:

( ) Male

( ) Female

( ) Other

6) Have you ever had contact with dairy farms?

( ) Yes

( ) No

7) What’s your profession?

.....................................................................

8) If you are a student, what is your field of study?

......................................................................

9) Where do you live?

City: ..........................................................................

State: ........................................................................

10) Do you live in urban or rural area?

( ) Urban

( ) Rural

( ) Both

11) How often do you consume dairy products (per week)??

...............

12) Have you ever heard about animal welfare?

( ) No

( ) Yes

13) In your opinion, the conditions of animal welfare in the dairy supply chain are:

Very bad 1 2 3 4 5 Very good

14) What is your level of concern about animal conditions in the dairy supply chain?

Very low 1 2 3 4 5 Very high

15) What is your level of agreement for each statement below’?

Strongly disagree Disagree Neutral Agree Strongly agree I do not know

1 2 3 4 5

Most dairy farmers focus too much on management instead of their animals

Most dairy farmers consider their animals too much as means of production

Most dairy farmers solely treat animals properly as long as it is financially beneficial

Most dairy farmers economize on animal welfare

Cattle on farms have quite a good life

Most dairy farmers give their animals a comfortable life

Most dairy farmers care about their animals

Most dairy farmers are in daily contact with their animals

Humans are allowed to use cattle for consumption

I feel guilty when I consume dairy products

16) In your opinion, dairy farmers in Brazil provide more animal welfare conditions to their animals compared to American and European farmers?

Strongly disagree 1 2 3 4 5 Strongly agree

17) What is your level of knowledge about the dairy supply chain?

( ) None;

( ) I kind of know it;

( ) I know it very well

18) What is your level of knowledge about animal welfare regulations?

( ) None;

( ) I kind of know it;

( ) I know it very well
